# Analysis of risk factors for anastomotic leakage after lower rectal Cancer resection, including drain type: a retrospective single-center study

**DOI:** 10.1186/s12876-020-01462-1

**Published:** 2020-09-25

**Authors:** Tetsushi Kinugasa, Sachiko Nagasu, Kenta Murotani, Tomoaki Mizobe, Takafumi Ochi, Taro Isobe, Fumihiko Fujita, Yoshito Akagi

**Affiliations:** 1 Department of Surgery, School of Medicine, Kurume University, 67 Asahi Machi, Kurume City, Fukuoka, Japan; 2 Biostatistics Center, Graduate School of Medicine, Kurume University, 67 Asahi Machi, Kurume City, Fukuoka, Japan

**Keywords:** Lower rectal cancer, Low anterior resection, Postoperative leakage, Drain type

## Abstract

**Background:**

We investigated the correlations between surgery-related factors and the incidence of anastomotic leakage after low anterior resection (LAR) for lower rectal cancer.

**Methods:**

A total of 630 patients underwent colorectal surgery between 2011 and 2014 in our department. Of these, 97 patients (15%) underwent LAR and were enrolled in this retrospective study. Temporary ileostomy was performed in each patient.

**Results:**

Anastomotic leakage occurred in 21 patients (21.7%). Univariate analysis showed a significant association between operative duration (*p* = 0.005), transanal hand-sewn anastomosis (*p* = 0.014), and operation procedure (*p* = 0.019) and the occurrence of leakage. Multivariate regression reanalysis showed that underlying disease (*p* = 0.044), transanal hand-sewn anastomosis (*p* = 0.019) and drain type (*p* = 0.025) were significantly associated with the occurrence of leakage. The propensity-score analysis showed that closed drainage were 6.3 times more likely to have anastomotic leakage than open drainage in relation to the amount of postoperative drainage (ml), according to the inverse probability of treatment-weighted analysis.

**Conclusions:**

Our results indicate that underlying disease, transanal hand-sewn anastomosis, and closed drain may be a risk and predictive factors for anastomotic leakage after LAR for lower rectal cancer. The notable finding was that closed drainage was related to the occurrence of anastomotic leakage and closed drainage was correlated with less volume of postoperative drain discharge than open drain.

## Background

Anastomotic leakage is a major postoperative complication after low anterior resection (LAR) for lower rectal cancer. Despite technical improvements and surgical device developments, double-stapling anastomosis with circular staples and transanal anastomosis is relatively difficult. The incidence of anastomotic leakage after LAR is 3–27% [1, 2]. Anastomotic leakage significantly increases postoperative morbidity, requiring a prolonged hospital stay and, in some patients, further surgical procedures [3], all of which affect patients’ quality of life. In advanced cancer patients with metastatic lymph nodes, adjuvant postoperative chemotherapy may be delayed, which could lead to an increased recurrence rate and a poor prognosis. We previously experienced cases of persistent anastomotic leakage after we changed from open to closed drainage after LAR for lower rectal cancer.

In this study, we investigated the correlations between surgery-related factors, including the type of drain, and the prevalence of anastomotic leakage after LAR for lower rectal cancer. Our previous experience suggested that the type of drain may be related to the frequency of postoperative complications. By clarifying these risk factors, we can improve patients’ outcomes by preventing the occurrence and severity of anastomotic leakage.

## Methods

Between 2011 and 2014, 630 patients underwent colorectal surgery in our department; among these, 149 patients had rectal cancer, excluding rectosigmoid cancer. This retrospective study included all 97 patients who underwent LAR (including intersphincteric resection and total colectomy) at our hospital from 2011 to 2014. Temporary ileostomy was performed in all patients, and no patients received preoperative chemoradiation. Surgeons in our department chose closed drainage or open drainage depending on the characteristics of the operation. We insert a drain in all cases of LAR. However, it is up to the surgeon to decide which drain to use. Informed consent was obtained from each patient before surgical resection, and the Institutional Review Research Committee for Human Subjects at Kurume University Hospital approved the study (no. 18197). Written approval consent was obtained from all the participant patients enrolled in this study. The data were accessed under the administrative permission.

The following data were extracted from the clinical records: sex, age, underlying disease (namely, diabetes mellitus, hypertension, coronary artery disease, and renal dysfunction), body mass index (BMI), stage, preoperative albumin value, operation duration, blood loss volume, anastomosis method, lateral lymph node dissection, type of drainage, drainage volume, and occurrence of leakage. The drainage volume was defined as the total amount drained from the day of surgery until the day of drain removal, according to patients’ medical records. The datasets used and/or analyzed during the current study are available from the corresponding author on reasonable request.

The Clavien–Dindo classification system [4] was used to define leakage and included Grade I complications. We confirmed anastomotic leakage with a digital examination, anoscopy findings, and enema imaging with an iodinated contrast agent.

**Fig. 1:**
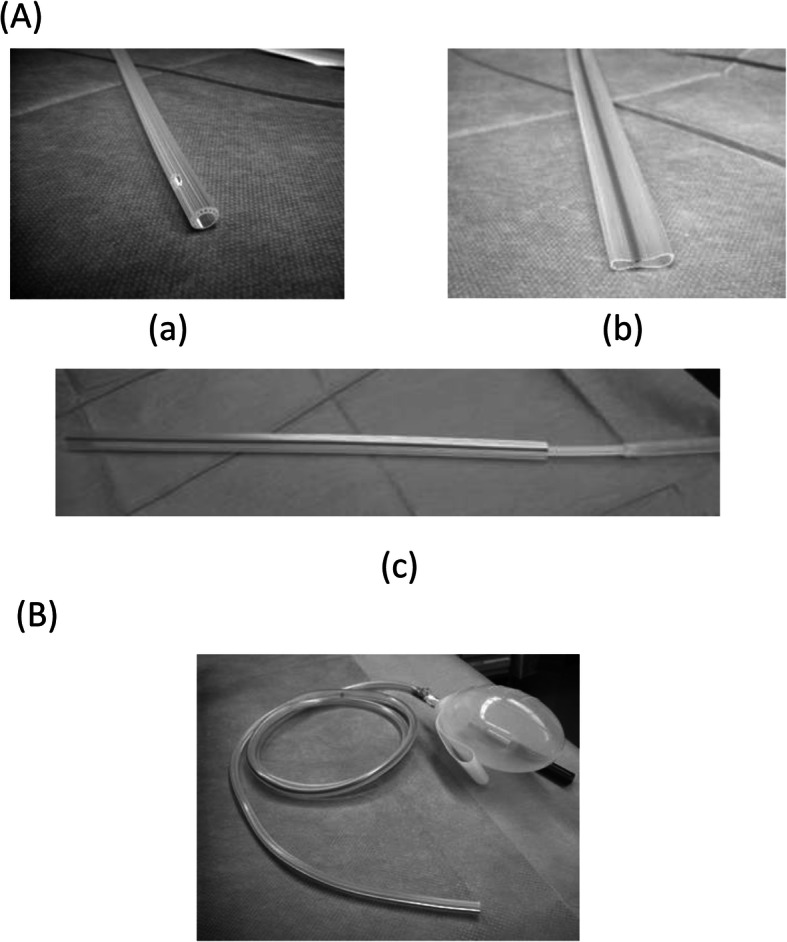
**A** Open drains. **a** Duple drain (6 Fr.); (**b**) Penrose drain (12 Fr.); (**c**) combining (**a**) and (**b**) into a single drain. **B** Closed drain. J-VAC drainage system (19 Fr.) (Johnson & Johnson, New Brunswick, NJ)

The drain was classified as one of two types: open (Group O) or closed (Group C). Patients in Group O had both a 6-Fr. duple drain (Kaneka Medical Products, Osaka, Japan) and a 12-Fr. Penrose drain (Fuji Systems Corp., Tokyo, Japan), whereas those in Group C had a 19-Fr. J-VAC drainage system (Johnson & Johnson, New Brunswick, NJ) (Fig. 1). In all patients, we inserted the drains around the anastomosis site.

### Statistical analysis

All variables are presented as mean with standard deviation or as number and percentage. Independent-samples t-tests were used to evaluate differences between both groups. Logistic regression analyses were performed to explore the risk factors associated with the presence or absence of leaks. Multivariate logistic models were developed using the backward selection method. A propensity-score analysis was performed to confirm the effect of drain type on leakage. Comparative analysis of leakage between the open drain and closed drain was performed both unadjusted and adjusted with propensity score, respectively. The propensity score was assessed by 5 different factors: basal disease, BMI, operation time, anastomosis, and operation. The main analysis used inverse probability of treatment weighting (IPTW). Although the population was small, propensity-score matching was used for sensitivity analysis. The statistical software package SAS ver. 9.4 (SAS Institute Inc., Cary, NC, USA) was used to perform all statistical analyses in this study, and all *p*-values of less than 0.05 were considered statistically significant.

## Results

### Patients’ characteristics

Table 1 summarizes the baseline characteristics of all the participate patients enrolled in this study. The median age was 64.2 years (range, 34–83 years); 76 patients (78.4%) were men, and 21 (21.6%) were women. The median BMI was 22.6 kg/m^2^. An open- drain was used in 56 patients (57.7%) and a closed drain in 41 patients (42.3%). The average drainage volume was 765 ml and the average preoperative albumin value was 3.93 g/dL. Fifteen patients (15.5%) underwent lateral lymph node dissection, whereas 82 patients (84.5%) did not. Forty-four patients (45.4%) had underlying disease (with duplicate cases), and 53 patients (54.6%) had no underlying disease.

**Table 1:** Background Clinical Characteristics of Enrolled Patients

	(*n* = 97)
Age (years: mean ± SD)	64.2 ± 10.76
Sex (male/female)	76 / 21
BD (positive / negative)	44 / 53
BMI (kg/m^2:^ mean ± SD)	22.6 ± 3.45
Blood loss (mls: mean ± SD)	316.0 ± 386.37
Operative duration (mins: mean ± SD)	363.0 ± 95.80
Anast (DST / HS)	59 / 38
Drain (open / close)	56 / 41
Alb (g/dl: mean ± SD)	3.93 ± 0.46
Drainage volume (mls: mean ± SD)	765.0 ± 451.36
LLD (+ / -)	15 / 82
Operation (CAA, ISR / LAR, uLAR)	38 / 59
Stage (I + II / III + IV)	58 / 39

*BD* basal disease, *BMI* body mass index, *Anast* anastomosis, *DST* double-stapling technique, *HS* hand-sewn, *LLD* lateral lymph node dissection, *CAA* coloanal anastomosis, *ISR* intersphincteric resection, *uLAR* ultra-low anterior resection, *Alb* albumin

We performed the following surgical procedures. LAR (in which the anastomosis was located on the anal side of the peritoneal reflection), ultralow anterior resection (u-LAR: a sphincter-saving procedure for very low-lying rectal cancers, in which anastomosis is performed with the double-stapling technique), coloanal anastomosis (CAA: a sphincter-saving procedure for very low-lying rectal cancers, in which hand-sewn anastomosis is performed), and intersphincteric resection (ISR) were performed in 45, 14, six and 32 patients, respectively. Fifty-nine patients (60.8%) underwent the double-stapling technique, and 38 patients (39.2%) underwent a hand-sewn technique for the anastomosis.

### Anastomotic leakage

Because patients with rectal cancer are more likely to have postoperative leakage than those with colon cancer, assessment of leakage was performed in all patients in the study. In the postoperative period after LAR, we evaluate the anastomosis with anoscopy every day. We also check the drain discharge properties until drain removal. In this study, the onset of postoperative leakage ranged from day 4 to day 8 after surgery. Anastomotic leakage occurred in 21 patients (21.7%); none developed a retrograde infection. The leakage-positive group included 21 patients, and the leakage-negative group included 76 patients (Table 2). No significant difference was found between the groups in terms of age, sex, underlying disease, BMI, intraoperative blood loss volume, preoperative albumin, or lateral lymph node dissection. Although not significantly different, the drainage volume was lower in Group C compared to the one in Group O. Significant differences were observed between both groups in operation duration (*p* = 0.003), anastomosis method (*p* = 0.023), and surgical procedure (*p* = 0.023).

**Table 2:** Background Clinical Characteristics of Patients with versus without Leakage

	Leakage		
	Positive (*n* = 21)	Negative (*n* = 76)	*p*-value
Age (years: mean ± SD)	65.81 ± 8.80	63.737 ± 11.2	0.437
Sex (Male/Female)	17 / 4	59 / 17	1
BD (Positive / Negative)	13 / 8	31 / 45	0.136
BMI (kg/m^2:^ mean ± SD)	23.51 ± 2.40	22.38 ± 3.67	0.185
Blood loss (mls: mean ± SD)	325.71 ± 378.3	313.89 ± 388.6	0.886
OP time (mins: mean ± SD)	418.04 ± 89.7	348.01 ± 92.3	0.003
Anast (DST / HS)	8 / 13	51 / 25	0.023
Drain (Open / Close)	9 / 12	47 / 29	0.139
preoperative Alb (g/dL: mean ± SD)	3.91 ± 0.42	3.94 ± 0.48	0.853
Drainage volume (mls: mean ± SD)	688.95 ± 449.9	785.68 ± 452.3	0.387
LLD (+ / -)	3 / 18	12 / 64	1
OP (CAA, ISR / LAR, uLAR)	13 / 8	25 / 51	0.023
Stage (I + II / III + IV)	11 / 10	47 29	0.460

*BD* basal disease, *BMI* body mass index, *OP* operation, *Anast* anastomosis, *DST* double-stapling technique, *HS* hand-sewn, *LLD* lateral lymph node dissection, *CAA* coloanal anastomosis, *ISR* intersphincteric resection, *uLAR* ultra-low anterior resection, *Alb* albumin

Table 3 shows the results of logistic regression analysis of the risk factors for leakage in patients who underwent LAR. In univariate analysis, operative duration (*p* = 0.005), transanal hand-sewn anastomosis (*p* = 0.021), and operation procedure (*p* = 0.019) were significantly associated with the occurrence of leakage after LAR. Besides, the leakage incidence was higher among patients with long operative duration for LAR and among those who underwent transanal hand-sewn anastomosis. In multivariate analysis, underlying disease (hazard ratio [HR]: 3.258, 95% confidence interval [CI]: 1.032–10.283; *p* = 0.044), transanal hand-sewn anastomosis (HR: 5.07, 95% CI: 1.31–19.632; *p* = 0.019), and drain type (HR: 4.311, 95% CI: 1.2–15.484; *p* = 0.025) were significantly correlated with the occurrence of leakage in patients who underwent LAR. Leakage after LAR occurred more commonly in patients with underlying disease, in those who underwent transanal hand-sewn anastomosis, and in those with closed drainage.

**Table 3:** Univariate and Multivariate Analyses of Leakage in Patients with Low Anterior Resection

Factor	OR	95%CI	*P* value	OR	95%CI	*P* value
Sex						
Male	1					
Female	0.817	0.242–2.754	0.744			
Age /years	1.019	0.972–1.069	0.434			
Basal disease	2.359	0.874–6.364	0.090	3.258	1.032–10.283	0.044
BMI	1.099	0.955–1.265	0.186			
Bood loss (mls)	1	0.999–1.001	0.885			
Operatiive duration (mins)	1.008	1.002–1.013	0.005	1.007	1–1.013	0.053
Anast						
DST	1			1		
Hand sewn	3.521	1.289–9.617	0.014	5.07	1.31–19.632	0.019
Drain						
Open	1			1		
Closed	2.161	0.811–5.76	0.124	4.311	1.2–15.484	0.025
preoperative Alb	0.905	0.321–2.558	0.851			
Discharge volume mls	1	0.998–1.001	0.384			
LLD performed	0.889	0.226–3.494	0.866			
Stage						
I + II	1					
III + IV	1.473	0.557–3.9	0.435			
OP						
CAA/ISR	1					
LAR/uLAR	0.302	0.111–0.822	0.019			

*Anast* anastomosis, *LLD* lateral lymph node dissection, *BMI* body mass index, *DST* double-stapling technique, *CAA* coloanal anastomosis, *OP* operation, *ISR* intersphincteric resection, *uLAR* ultra-low anterior resection, *Alb* albumin

We performed a propensity-score analysis to confirm these findings. Because the population was imbalanced, we used IPTW in the main analysis. Although the population was small, we used propensity-score matching. Table 4 shows the propensity-score analysis results (unadjusted HR: 2.161, *p* = 0.124; adjusted with IPTW, HR: 6.315, *p* < 0.001; propensity-score matching: HR: 5, *p* = 0.174). The IPTW analysis revealed a significant difference between results in patients with an open-drain versus a closed drain. Closed drainage was associated with a 6.315 times higher incidence of postoperative leakage than open drainage. Table 5 shows a significant difference in the average drainage volume (*p* < 0.001), which was 954 ml in Group O and 507 ml in Group C. Table 6 shows the percentage of each operation procedure for each type of drain. In the group with open-drain (*n* = 56), CAA/ISR was performed in 28 cases (50.0%) and LAR/u-LAR was performed in 28 cases (50.0%). In the closed-drain group (*n* = 41), CAA/ISR was performed in 10 cases (24.4%) and LAR/u-LAR was performed in 31 cases (75.6%). In the open-drain group, the operation method was evenly divided, whereas, in the closed-drain group, LAR/u-LAR was performed in 2/3 of procedures.

**Table 4:** Propensity-Score Analysis

Method	Category	n	OR	95% CI		*P* value
Unadjusted	Open	97	1			
	Closed		2.161	0.811	5.76	0.124
IPTW	Open	96	1			
	Closed		6.315	3.008	13.256	< 0.001
Matching	Open	32	1			
	Closed		5	0.492	50.831	0.174

*IPTW* inverse probability of treatment-weighted

**Table 5:** Average Drained Volume Analysis

Type of drain		
Group-O	56 cases (57.7%)	
Group-C	41 cases (42.3%)	
Average drained volume (mL)		
Group-O	954 ± 437.4	*p* < 0.001
Group-C	507 ± 328.0	

*O* open drain, *C* closed drain

**Table 6:** Relationship between Drain Type and Operative Procedure

Type of drain	Operation	
Group-O (56 cases)	CAA/ISR	28 cases (50.0%)
	LAR/u-LAR	28 cases (50.0%)
Group-C (41 cases)	CAA/ISR	10 cases (24.4%)
	LAR/u-LAR	31 cases (75.6%)

*CAA* coloanal anastomosis, *ISR* intersphincteric resection, *uLAR* ultra-low anterior resection, *O* open drain, *C* closed drain

## Discussion

Anastomotic leakage is a major postoperative complication after lower rectal surgery and is associated with high postoperative morbidity and mortality, functional defects, and poor oncological outcomes [5, 6]. Several risk factors have been reported for anastomotic leakage after open LAR [7–11] and, after laparoscopic LAR [12–21]. However, the devices and techniques used for laparoscopic LAR differ from those used in open LAR, suggesting that the risk factors for anastomotic leakage may also differ between the two different surgical approaches. Besides, the anastomotic level, number of linear staples, sex, smoking habits, alcohol intake, previous abdominal surgery, preoperative chemoradiotherapy, tumor location and grade, stage, operative duration, blood loss volume, transfusion, and precompression before firing are reported as a potential risk and predictive factors for anastomotic leakage after LAR. In the present study, our investigation of potential risk factors suggests that the presence of underlying disease, the use of transanal hand-sewn anastomosis, and the use of closed drains may increase the risk for anastomotic leakage.

In previous studies, intraoperative blood loss volume was reported as an independent risk factor for anastomotic leakage [17–19, 21, 22]. In the present study, we found no significant association between blood loss volume and anastomotic leakage, suggesting that anastomotic leakage did not occur directly because of bleeding and that intraoperative blood loss volume was likely to be a surrogate for surgical complication.

The duration of surgery was reported as a risk factor in some studies [23–25]. Our study confirmed that patients with a longer surgical duration had a higher incidence of anastomotic leakage and this may be caused by lower surgical skill or poor exposure of the surgical field secondary to pelvic stenosis or a bulky tumor. Besides, decreased blood perfusion caused by prolonged anesthesia may increase the risk of anastomotic leakage.

Sánchez-Guillén et al. reported the perioperative risk factors for anastomotic leakage and 60-day morbidity and mortality after ileocolic anastomosis (stapled vs hand-sewn). The authors’ multivariate regression analysis showed the following independent risk factors for major anastomotic leak: male sex (*P* = 0.014, odds ratio [OR]: 2.9), arterial hypertension (*P* = 0.048, OR: 2.29), and perioperative transfusion (*P* < 0.001, OR: 2.4 per liter). In this study, the overall 60-day complication rate in that study was 27.3%. The complication rate was 31.3% in male vs. 22.3% among female patients (*P* = 0.020, OR: 1.7), diabetes (*P* = 0.030, OR: 2.0), smoking habit (*P* = 0.040, OR: 1.8), and perioperative transfusion (P < 0.001, OR: 3.3 per liter) were independent risk factors for postoperative morbidity [26]. These results are consistent with our underlying disease results, which suggest that the presence of the underlying disease is associated with anastomotic leakage.

Several studies have reported that tumor location and its distance from the anal verge are risk factors for anastomotic leakage after LAR [13–17, 20]. In a series of 156 patients who underwent LAR without double stapling, Choi et al. reported that the anastomotic leakage rate was 10 times higher when the anastomotic region was located within 5 cm of the anal verge [15]. Besides, tumor location and distance from the anal verge may reflect the technical difficulty and affect anastomotic tension and blood supply. In the present study, the multivariate analysis showed a statistically significant difference in leakage occurrence between double-stapling and hand-sewn anastomosis. Therefore, we concluded that both double-stapling and hand-sewn anastomosis were likely to be risk factors for anastomotic leakage after LAR.

Our results showed that the type of drain was related to anastomotic leakage after LAR. To our knowledge, this result has not been reported previously and is considered an important finding. An open-drain can be used for effective long-term drainage, but the possibility of retrograde infection is a concern. In contrast, a closed drain is less likely to be associated with retrograde infection, but the obstruction is a problem. Although some reports have described the risk of retrograde infection in patients with open drainage [27–29], none has reported the related frequency or any diagnostic criteria. In the present study, no retrograde infection occurred in patients with open drainage. A peritoneal defect is sometimes present within the pelvis after rectal resection. This loss of peritoneum decreases reabsorption of effusion and increases the risk of infection, predisposing to abscess formation. There is a strong possibility that these conditions lead to leakage.

A single surgeon had the following experience in consecutive ISR cases. The surgeon generally used an open drain for ISR cases. At one point, he changed to a closed drain in two consecutive ISR cases. Those two patients developed leakage. Based on that experience, the surgeon switched back to open drains for ISR cases and subsequently no leakage occurred. Because efficient fluid drainage is important, we consider it necessary to carefully consider which type of drain to use in digestive surgery. Surprisingly, in the propensity-score analysis, patients with closed drainage had a 6.315 times higher risk of postoperative leakage than those with open drainage. This finding is impressive and important, and statistically meaningful.

Our study has a few limitations. The sample size was small and the study design was a retrospective study conducted at a single institution. The rate of anastomotic leakage in this study was slightly higher (21.7%) than that in other studies. This higher percentage may be attributed to the fact that many of the patients in this study had advanced disease. Moreover, many patients had Rb-positive lesions, which may have caused selection bias. Moreover, we excluded patients who received preoperative chemotherapy or chemoradiotherapy because of our department’s treatment policy. Since the number of cases in this analysis is not sufficient, confirmation by real-world data in a population with a larger number of cases is required [30]. A prospective study involving multiple institutions with a unified definition of anastomotic leakage and consistent procedures is needed. However, to our knowledge, no studies have collected and analyzed drainage data in patients undergoing lower rectal surgery; therefore, our findings are noteworthy.

## Conclusion

Anastomotic leakage is a multifactorial complication after LAR. Patients’ characteristics cannot be changed, but novel devices and technical improvements could prevent this complication. In this study, we demonstrated that in patients with anastomotic leakage after LAR, leakage frequency was higher in those with underlying disease, who underwent transanal hand-sewn anastomosis, and in those with closed drainage. The study findings suggest that it is essential to determine the need for a drain and to select the drainage method after a comprehensive assessment of the surgical procedure and the patient’s condition.

## Data Availability

After publication of the primary findings, the de-identified and completely anonymized individual participant-level dataset will be posted on the UMIN-ICDR website (http://www.umin.ac.jp/icdr/index-j.html) so that it can be accessed by qualified researchers.

## Abbreviations

BMI: Body mass index
LAR: Low anterior resection
IPTW: Inverse probability of treatment-weighted
HR: Hazard ratio
CI: Confidence interval
OR: Odds ratio
